# Malignant adenomyoepithelioma of the breast

**DOI:** 10.1186/s40792-020-00881-2

**Published:** 2020-05-29

**Authors:** Kazuki Moro, Eiko Sakata, Asa Nakahara, Hideki Hashidate, Emmanuel Gabriel, Haruhiko Makino

**Affiliations:** 1 Department of Breast Surgery, Niigata City General Hospital, 463-7 Shumoku, Chuo-ku, Niigata, 950-1197 Japan; 2 Department of Pathology, Niigata City General Hospital, 463-7 Shumoku, Chuo-ku, Niigata, 950-1197 Japan; 3 Department of Pathology, Brain Research Institute, Niigata University, Niigata City, 950-8585 Japan; 4 Department of Surgery, Mayo Clinic, Jacksonville, FL 32224 USA; 5 Makino Breast Clinic, Niigata, 950-0861 Japan

**Keywords:** Differentiation, Ductal spread, Hematogenous spread, Malignant adenomyoepithelioma, Surgical margin

## Abstract

**Background:**

Adenomyoepithelioma (AME) of the breast is a very rare tumor and is generally considered to be benign. However, some show malignant transformation, which results in local recurrences or distant metastases. The morphological features of AME that might predict malignant potential have not been elucidated. Moreover, there is also no established multidisciplinary treatment for malignant AME aside from complete excision at an early stage.

**Case presentation:**

A 64-year-old female diagnosed with AME of the left breast underwent lumpectomy. The surgical margins were negative. Six months after the operation, however, malignant AME recurred locally in the left breast. MRI showed multiple masses, which invaded the skin. A left mastectomy with axillary lymph node dissection was performed. Additional areas of AME were found in about one third of the entire breast. Eight months after the mastectomy, lung metastases were detected. She underwent chemotherapy with fluorouracil, epirubicin, and cyclophosphamide (FEC) for 9 cycles with little response. Lung metastasectomy was performed. Nine months after lung metastasectomy, the metastases were widespread to the brain, heart, and kidney; she subsequently died 2 months later.

**Conclusions:**

Malignant AME has various morphological features, and in this report, we characterize new findings from both imaging and pathology/autopsy. Malignant potency is related to the tumor size, tumor appearance, and mitoses, even if only a few. Given that ductal spread is one of the morphological features of malignant AME, it is of paramount importance to assess the surgical margins.

## Background

Adenomyoepithelioma (AME) of the breast is a rare disease characterized by a bicellular pattern consisting of both ductal and myoepithelial cells [1, 2]. While most of AMEs of the breast are benign with good prognosis, some have shown malignant transformation [3, 4]. Malignant AME is difficult to differentiate from other benign diseases such as intraductal papilloma, tubular adenoma, and sclerosing adenosis. Moreover, malignant AME has a strong potential for local recurrence and distant metastasis to sites including the lungs [4], thyroid gland [5], bone [6], and brain [3].

Since the morphological features of AME that could predict the malignant potency have not been elucidated, the tumors which seem to be benign have the possibility of changing into malignant tumors. Our case is atypical in that we describe new morphological features not previously reported. Thus, our case of malignant AME is of interest not only for its rarity, but also for the aspects of the morphological features.

## Case presentation

**Fig. 1:**
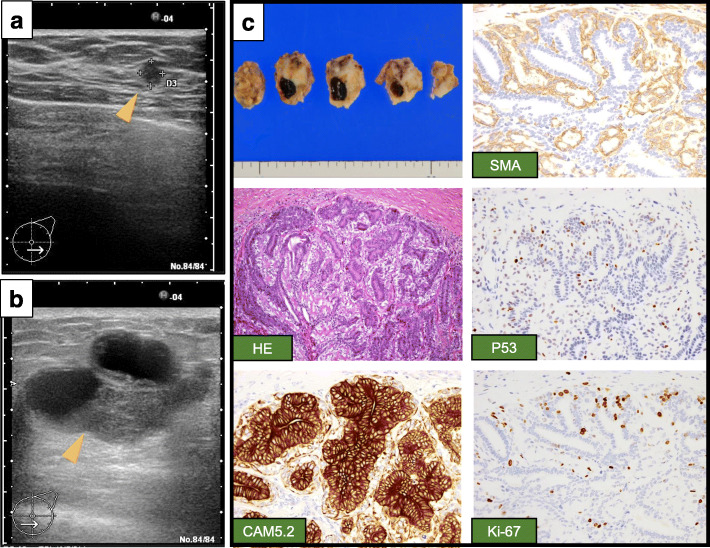
Ultrasonographic examination from the first consultation to the primary operation. **a** An ultrasonography (US) scan of the breast demonstrated a low-density area measuring 4.9 × 5.1 × 4.2 mm (orange arrowhead). **b** An US scan of the breast demonstrated a low-density area measuring 26.1 × 22.6 × 26.8 mm (orange arrowhead). **c** Histopathological findings of the tumor at the primary operation. Although mitotic figures were present slightly, there was no proliferative lesion or ductal invasion. The tumor was consisted of both epithelial cells, which was positive for CAM 5.2, and myoepithelial cells, which was strongly positive for α-smooth muscle actin (SMA). Ki-67 labeling index (Ki-67) and P53 were weakly positive. × 200

A 64-year-old female with no significant past medical history was referred to our institution after new microcalcifications were identified in the left breast on screening mammography. Diagnostic ultrasonography (US) showed a 4.9 × 5.1 × 4.2 mm low echoic mass on the left between external-inferior and internal-inferior quadrants (Fig. 1a). Only duct papillomatosis was found on core needle biopsy. This was found to be concordant, and she was treated with observation.

Two and a half years after the first consultation, she palpated a mass at the same location. A new US highlighted a larger 26.1 × 22.6 × 26.8 mm low echoic mass (Fig. 1b). Benign adenomyoepithelioma (AME) was identified on core needle biopsy. As the patient was a candidate for breast conservation, lumpectomy was performed. The histological analysis revealed a benign AME with few mitotic figures measuring 31 × 27 × 21 mm. All surgical margins were negative (Fig. 1c). The tumor consisted of both epithelial cells positive for CAM 5.2 and myoepithelial cells positive for α-smooth muscle actin (SMA) (Fig. 1c).

**Fig. 2:**
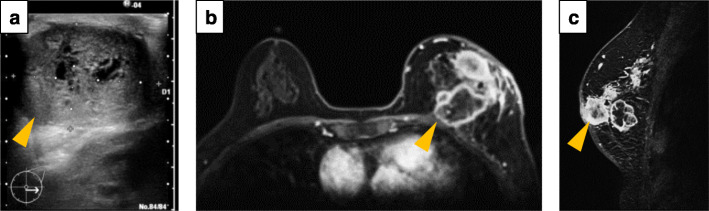
The data of the secondary operation. **a** An ultrasonography (US) scan of the breast demonstrated a scale out size of low echoic mass (orange arrowhead). **b** A magnetic resonance imaging (MRI) showed some tumors including internal necrosis with ductal spread (orange arrowhead). The pectoralis muscle invasion was suspected. **c** A sagittal sequence of MRI scans showed the wide ductal spread of malignant adenomyoepithelioma (AME), which invaded the skin (orange arrowhead)

**Fig. 3:**
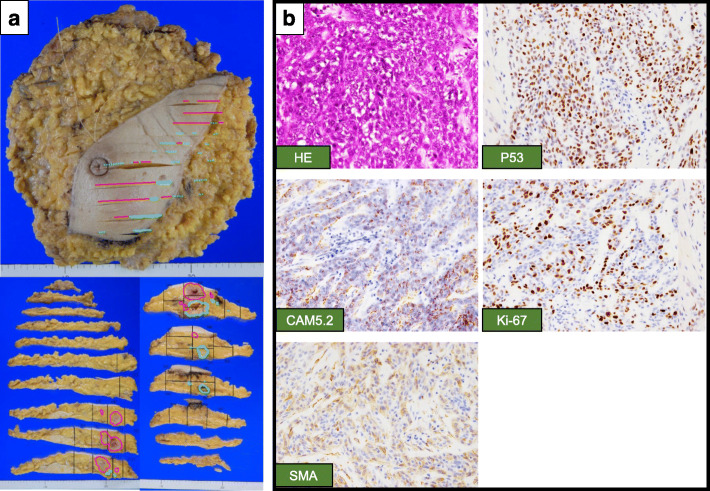
Histopathology of the malignant adenomyoepithelioma. **a** Gross specimen of malignant AME. Red markers show malignant lesions, and blue markers show ductal lesions. The size of tumor was 110 × 105 × 55 mm. **b** Histopathology of the malignant AME. The tumor was consisted of both epithelial cells, which was positive for CAM 5.2, and myoepithelial cells, which was positive for α-smooth muscle actin (SMA). Ki-67 labeling index (Ki-67) was 44%. The myoepithelial cells were strongly positive for P53. × 200

Six months after the primary operation, she noticed a mass at the same location again. Diagnostic US highlighted a 34 × 26 mm hypoechoic mass along the left lumpectomy cavity (Fig. 2a). A computed tomography (CT) scan of the chest, abdomen, and pelvis showed no signs of distant metastasis. MRI showed multiple masses, which invaded the skin. Pectoralis muscle invasion was also suspected (Fig. 3b, c).

We therefore performed a left mastectomy and axillary lymph node dissection. Pathological examination showed a malignant AME with multiple AME tumors collectively measuring 110 × 105 × 55 mm (Fig. 3a). The tumor consisted of both epithelial cells positive for CAM 5.2 and myoepithelial cells positive for SMA. Ki-67 labeling index (Ki-67) was 44%. The myoepithelial cells were strongly positive for P53 (Fig. 3b). Additional intraductal consistent with AME were spread about one third of the entire breast. Immunohistochemical analysis showed that these lesions were also consistent with AME. Resected lymph nodes (0/26) were negative.

**Fig. 4:**
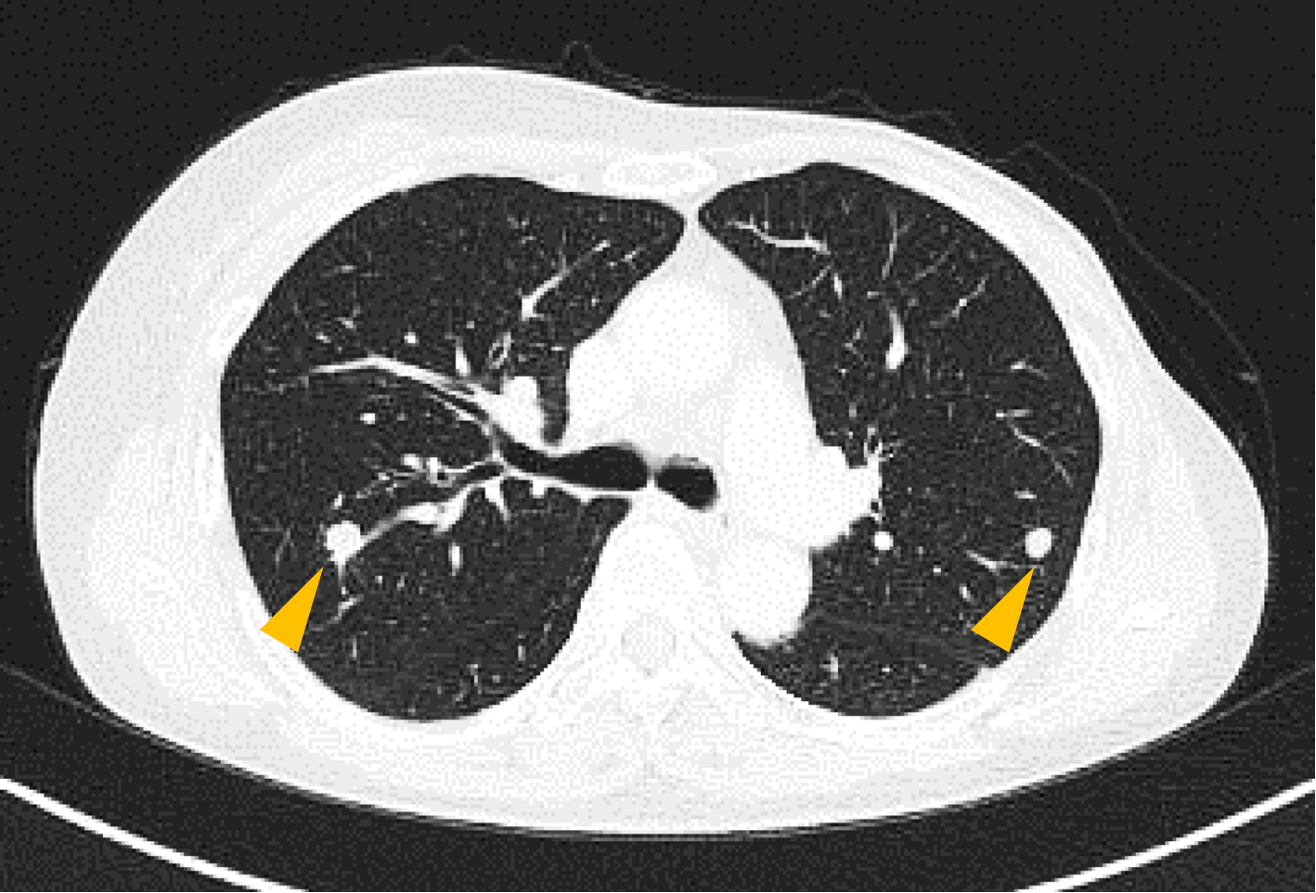
Images of computed tomography (CT) at diagnosis showing lung metastasis. CT revealed two nodular masses located in the patient’s left upper and right upper lobes of the lungs (orange arrowheads)

Eight months after the secondary operation, chest X-ray and CT revealed two nodular masses located in her left upper (measuring 8 × 7 mm) and right upper lobes of the lungs (measuring 10 × 7 mm) (Fig. 4). She underwent chemotherapy with fluorouracil, epirubicin, and cyclophosphamide (FEC) for 9 cycles. Imaging after 9 cycles showed a partial reduction in the lung metastases and no other new distant metastasis. Due to the cardiotoxicity of epirubicin, we elected to perform pulmonary metastasectomies. A left upper lobectomy with video-assisted thoracic surgery (VATS) and subsequently a right segmentectomy (S2) were performed.

**Fig. 5:**
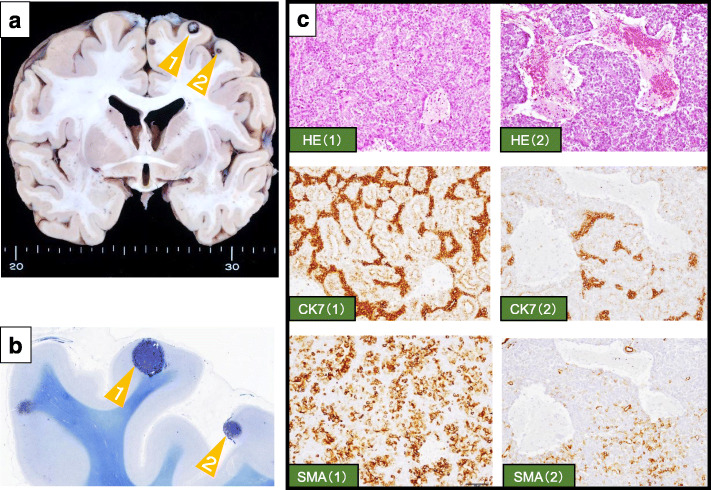
Images of autopsies. **a** Gross specimen of brain. There are some metastatic lesions (orange arrowheads). **b** Histopathological findings of the brain. The tissue of the brain metastases stained with Klüver-Barrera staining. **c** The brain metastases (in **a** number 1 and number 2) were positive for CK7 and α-smooth muscle actin (SMA) retrospectively. The epithelial component was increased in the metastatic lesions compared to the primary breast tumor

Nine months after the metastasectomy, however, she presented with right lower abdominal pain and dysuria. A whole-body CT scan demonstrated multiple lung metastases, right kidney metastasis, right adrenal metastasis, ovarian metastasis, and abdominal paraaortic node metastasis. She underwent salvage eribulin monotherapy. The whole-body CT after eribulin 3 cycles demonstrated that the diseases continued to progress. Two months later, she had expired. Autopsy demonstrated that the metastasis was widespread to the heart muscle, kidney, and brain (Fig. 5a–c).

## Discussion

The definition of malignant AME is not clearly defined. Nadelman et al. described 2 cases of metastases of histologically “benign” AME of the breast to the lung [7]. Some AME tumors appear benign but may contain cellular atypia or mitotic figures. Although morphological features of malignant transformation include nuclear atypia, increased mitotic activity, necrosis, and infiltrative growth pattern [8], there is no established reference to differentiate between benign and malignant AMEs. In our case, mitotic figures were present in the tumor at the primary operation. Considering the patient’s course, this may have provided a clue as to the malignant potential of her primary tumor. Tumor size is also one of the characteristics that may be related to potential malignancy. Patients with a primary tumor of ≥16 mm often presented with metastases [9]. Some papers have concluded that AMEs over 2 cm should be treated as malignant [10, 11]. In our case, the size of the tumor at the primary operation was 31 mm, leading to poor prognosis. In addition to the tumor size, tumor appearance is an important factor in prognosis. Generally, malignant AME has been described as a large stable mass [12], but our case of malignant AME showed multifocality within the breast. To the best of our knowledge, this represents an unusual presentation of AME. Other factors including mitotic figures, tumor size, and tumor appearance are also indicators of the malignant potential.

In managing the malignant AME, it is important to recognize their poor prognosis. It is known that P53 and Ki-67 are important prognostic factors. In comparison to immunohistochemical features of malignant AME, cases with higher expressions of P53 and Ki-67 were worse prognostic factors [13]. In our case, both of P53 and Ki-67 were high, leading to the observed poor prognosis. There are also no unified views whether hematogenous spread or lymphatic spread occurs in malignant AME, but metastases to the axillary lymph nodes are extremely rare [12]. The pattern of spread in our case suggests that metastatic malignant AME metastasizes mainly through the hematogenous route rather than lymphatic system.

In general, the biological behavior of tumors developing in mammary glands ranges from benign to malignant transformation of either the epithelial or the myoepithelial component or both. As portrayed by our case, the biological behavior of tumors is different between primary and recurrent lesions. Although all of the lesions consisted of both the epithelial component and the myoepithelial component, the proportions of the two components were different. The epithelial component was most abundant in the primary site (Fig. 1c) followed by the brain metastases (Fig. 5c), and least in secondary site, which was diagnosed as malignant AME (Fig. 3b). Moreover, the proportions of epithelial and myoepithelial cells were different among the brain metastases (Fig. 5c). Recognizing that heterogeneity between the proportions of epithelial and myoepithelial cells impacts treatment resistance [14], the increased proportion of the myoepithelial component compared to the epithelial component likely contributes to worse prognosis.

The treatment of malignant AME is not established except for complete excision at an early stage. Kihara et al. concluded that a complete local excision remains the only way to reduce the chance of local recurrence and distant metastases [4]. On the other hand, it remains unknown whether axillary lymph node sampling is necessary. Similar to surgical treatment, there is no effective adjuvant chemotherapy at present. Chemotherapy has been used in some malignant cases, but the majority of them are not effective [5, 6]. Lee et al. reported that eribulin had a beneficial effect on malignant AME of the breast with multiple hepatic, pleural, and abdominal wall metastases [15]. Neither complete resection of lung metastases nor chemotherapy including FEC and eribulin could control the malignant AME in our case.

Malignant AME can progress very aggressively as it did in the current case. Even if AME presents in a benign manner, it is important to assess the extent of the primary lesion by MRI and to consider wide surgical margins at the primary operation to perform complete resection as this may be the only potential option for a favorable outcome.

## Conclusions

Malignant AME has various morphological features, and we demonstrated unique findings from both imaging and pathology/autopsy. Even only a few mitotic figures should raise caution regarding the malignant potential of the tumor in addition to the size and appearance of AME. Considering that ductal spread is one of the more aggressive morphological features of malignant AME, it is of paramount importance to assess the surgical margin before resection and obtain widely negative surgical margins.

## Data Availability

Not applicable

## Abbreviations

AME: Adenomyoepithelioma
CT: Computed tomography
FEC: Fluorouracil, epirubicin, and cyclophosphamide
Ki-67: Ki-67 labeling index
MRI: Magnetic resonance imaging
SMA: α-Smooth muscle actin
US: Ultrasonography
VATS: Video-assisted thoracic surgery
